# RETRACTION: Effect of Hydrocolloid Dressings in the Management of Different Grades of Pressure Wound Ulcers in Critically Ill Adult Subjects: A Meta‐Analysis

**DOI:** 10.1111/iwj.70189

**Published:** 2025-01-12

## Abstract

**RETRACTION**: HuangR. , HuaZ. , LiL. , ZhouY. , XuY. , and ZhangT. , “Effect of Hydrocolloid Dressings in the Management of Different Grades of Pressure Wound Ulcers in Critically Ill Adult Subjects: A Meta‐Analysis,” International Wound Journal 20, no. 10 (2023): 3981–3989, 10.1111/iwj.14286.37434335 PMC10681538

The above article, published online on 11 July 2023, in Wiley Online Library (http://onlinelibrary.wiley.com/), has been retracted by agreement between the journal Editor in Chief, Professor Keith Harding; and John Wiley & Sons Ltd. Following an investigation by the publisher, all parties have concluded that this article was accepted solely on the basis of a compromised peer review process. In addition, the investigation found unattributed textual overlap between this article and another article by different authors (Kamińska, et al. 2020 [https://doi.org/10.3390/ijerph17217881]). The editors have therefore decided to retract the article. The authors did not respond to our notice regarding the retraction.

**RETRACTION**: R. Huang , Z. Hua , L. Li , Y. Zhou , Y. Xu , and T. Zhang , “Effect of Hydrocolloid Dressings in the Management of Different Grades of Pressure Wound Ulcers in Critically Ill Adult Subjects: A Meta‐Analysis,” International Wound Journal 20, no. 10 (2023): 3981–3989, 10.1111/iwj.14286.37434335 PMC10681538

The above article, published online on 11 July 2023, in Wiley Online Library (http://onlinelibrary.wiley.com/), has been retracted by agreement between the journal Editor in Chief, Professor Keith Harding; and John Wiley & Sons Ltd. Following an investigation by the publisher, all parties have concluded that this article was accepted solely on the basis of a compromised peer review process. In addition, the investigation found unattributed textual overlap between this article and another article by different authors (Kamińska, et al. 2020 [https://doi.org/10.3390/ijerph17217881]). The editors have therefore decided to retract the article. The authors did not respond to our notice regarding the retraction.

